# Fractional Photothermolysis Using a Time‐Gated Millisecond 1070 nm Fiber Laser

**DOI:** 10.1111/jocd.71089

**Published:** 2026-07-26

**Authors:** Michael Wang‐Evers, Aditi Maheshwari, Dilip Paithankar, Ilya V. Yaroslavsky, Gregory Altshuler, Dieter Manstein

**Affiliations:** ^1^ Department of Dermatology Harvard Medical School, Massachusetts General Hospital, Cutaneous Biology Research Center Boston Massachusetts USA; ^2^ IPG Medical, IPG Photonics Corporation Marlborough Massachusetts USA

**Keywords:** fractional, laser, nonablative, vascular

To the Editor,

Selective photothermolysis relies on wavelength and temporal selectivity, where light is preferentially absorbed by a target chromophore using pulse durations matched to its thermal relaxation time, enabling localized thermal injury [1]. In contrast, fractional photothermolysis achieves spatial selectivity by delivering energy in a pixelated pattern, creating microscopic treatment zones (MTZs) driven by water absorption [2]. Selective Fractional Photothermolysis (SFP) has previously been explored using 1064 nm Nd:YAG lasers, but tissue effects were limited by low water absorption and insufficient output power [3]. Although hemoglobin‐selective wavelengths are used for vascular targeting, they are constrained by melanin absorption and limited depth, whereas 1070 nm enables deeper penetration with a greater contribution from water absorption [4]. With the availability of high‐power fiber lasers, this approach can now be revisited. We evaluated a 1070 nm Ytterbium fiber laser as a nonablative fractional modality for generating spatially confined subsurface thermal lesions at depths potentially relevant to vascular lesions such as port‐wine stains [4, 5, 6].

The laser (YLPN‐2‐20 × 500–300, IPG Photonics) operated in continuous‐wave mode, with emission time‐gated by TTL control to produce 1–6 ms exposures. Energy delivery ranged from 0.5–3 J per exposure through a 50 μm focal spot and was applied in a 6 × 6 fractional array with 2 mm pitch spacing. Ex vivo porcine abdominal tissues, including nonpigmented skin, pigmented skin, and kidney tissue as a vascular‐rich comparison tissue, were frozen within 1 h of excision, thawed before use, maintained at approximately 32°C, hydrated with saline, irradiated, and stained with nitro‐blue tetrazolium chloride to visualize thermally denatured regions (Figure 1).

**FIGURE 1:**
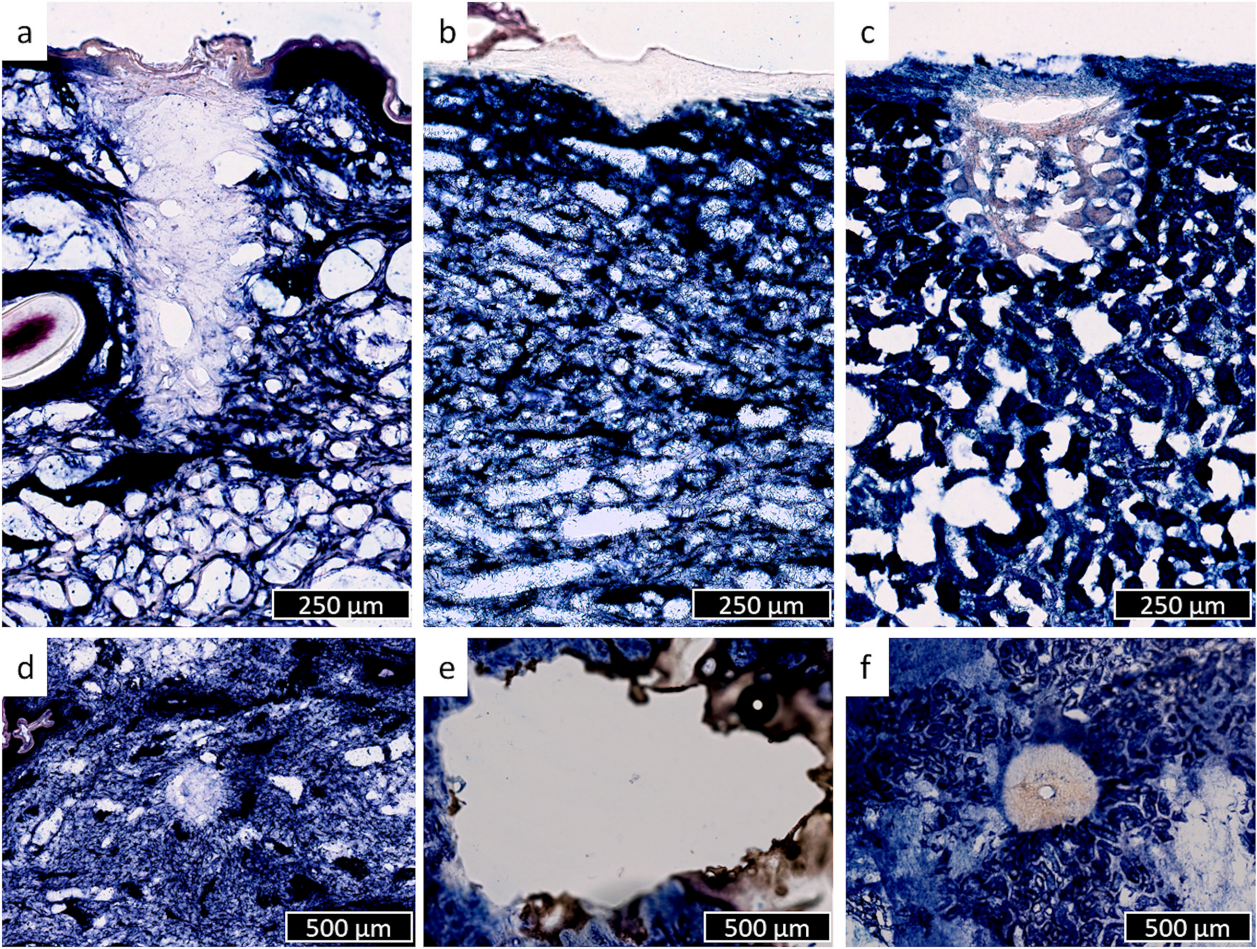
NBTC‐stained histological sections (vertical and horizontal) of 1070 nm laser‐induced thermal lesions in nonpigmented skin (a, d), pigmented skin (b, e), and kidney tissue (c, f) at 2 J per pulse. Epidermal ablation is evident in pigmented skin samples (b, e).

In nonpigmented skin, lesion depth increased from 518 to 780 μm as energy increased from 0.5 to 3 J, with lateral diameters of 186–274 μm (Figure 2). In pigmented skin, enhanced melanin absorption produced shallower (104–310 μm) but broader (531–1600 μm) lesions. These dimensions substantially exceeded the incident beam diameter, consistent with epidermal ablation, scattering, and lateral thermal diffusion after intense melanin absorption. In kidney tissue, lesions ranged from 126–358 μm in depth and 264–488 μm in diameter. These patterns are consistent with known chromophore absorption differences at 1070 nm, which influence the initial distribution of deposited energy; however, ultimate lesion depth and confinement are also limited by optical scattering and thermal diffusion. Because melanin and hemoglobin concentrations were not directly measured, these findings should be interpreted qualitatively. No lesion exceeded 1 mm in depth, suggesting that optical scattering and millisecond‐scale exposure durations limited vertical confinement [1, 4]. Nevertheless, these findings demonstrate that fractional photothermal lesions can be generated at 1070 nm. To our knowledge, this represents the first demonstration of spatially confined thermal lesion formation in skin within this wavelength range.

**FIGURE 2:**
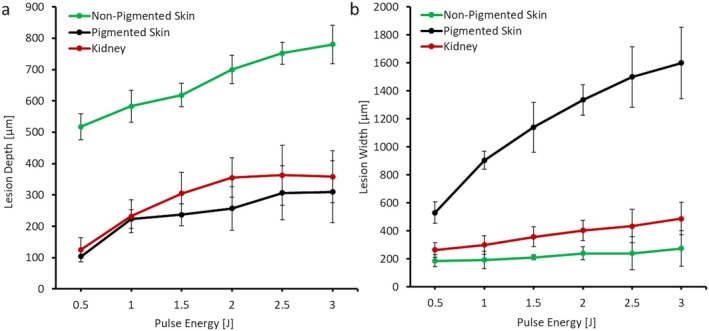
Graphical summary of lesion depth (a) and diameter (b) vs. pulse energy for pigmented skin, nonpigmented skin, and kidney tissue.

From a translational perspective, the required pulse energies (0.5–3 J) approach levels where clinical safety cannot be assumed and would likely require active cooling and optimized beam shaping. The millisecond‐scale exposures used here exceed the thermal relaxation times of many target chromophores and promote heat diffusion during energy delivery. Future systems should therefore employ higher peak powers and shorter pulse durations, on the order of tens to hundreds of microseconds, to improve thermal confinement and reduce collateral injury.

This proof‐of‐concept study supports reintroduction of SFP at 1070 nm as a framework for generating spatially confined thermal injury. Although this ex vivo study does not establish selective vascular targeting, the combination of deep penetration and fractional confinement may inform future nonablative systems for deep vascular lesions, including recalcitrant port‐wine stains and venous malformations. The response of pigmented tissue also suggests potential application to pigmentary lesions. Further in vivo studies incorporating perfusion, safety assessment, and vessel‐specific histology are needed to evaluate biological responses and therapeutic potential under physiological conditions.

## Funding

The authors have nothing to report.

## Ethics Statement

Ex vivo porcine skin and kidney tissue were utilized; no live animal or human participants were involved.

## Conflicts of Interest

IPG Photonics Corporation employs Dilip Paithankar, Ilya Yaroslavsky, and Gregory Altshuler. The company markets the laser system used in this study. The other authors declare no conflicts of interest.

## Data Availability

The data that support the findings of this study are available from the corresponding author upon reasonable request.
